# Modulation of Repeated Anodal HD-tDCS on Attention in Healthy Young Adults

**DOI:** 10.3389/fpsyg.2020.564447

**Published:** 2020-11-20

**Authors:** Hongliang Lu, Quanhui Liu, Zhihua Guo, Guangxin Zhou, Yajuan Zhang, Xia Zhu, Shengjun Wu

**Affiliations:** ^1^Department of Military Medical Psychology, Air Force Military Medical University, Xi’an, China; ^2^School of Basic Medical Sciences, Air Force Military Medical University, Xi’an, China

**Keywords:** transcranial direct current stimulation, HD-tDCS, attention, attention network, executive control, attention network test, stroop

## Abstract

High-definition transcranial direct current stimulation (HD-tDCS) is a valid brain stimulation technology to optimize cognitive function. Recent evidence indicates that single anodal tDCS session enhances attention; however, the variation in attention produced by repeated anodal HD-tDCS over a longer period of time has not been explored. We examined the modulation of attention function in healthy young participants (39 young adults) who received repeated HD-tDCS sustained for 4 weeks. The results showed a robust benefit of anodal HD-tDCS on executive control and psychomotor efficiency, but not on orienting, alerting, or selective attention (inhibition); the benefit increased successively over 4 weeks; and the enhancement on executive control of each week was significant compared to baseline in the anodal group. In addition, the subjects’ performances on the test of executive control and psychomotor efficiency gradually restored to the initial level in the sham group, which appeared obviously from week 3 (after 9 interventions), but the improvement of attention in the anodal group was persistent. We conclude that repeated anodal HD-tDCS provides a positive benefit on executive control and psychomotor efficiency and has obvious accumulative effect after 9 or more times intervention compared to sham HD-tDCS. Additionally, our findings might provide pivotal guidance for the formulation of a strategy for the use of repeated anodal HD-tDCS to modulate on attention function.

## Introduction

As a painless, reversible, and non-invasive brain stimulation technology, transcranial direct current stimulation (tDCS) is usually used to improve symptoms in individuals with neuropsychiatric disease, and, in recent years, this method has also been found to be effective in enhancing the cognitive performance of healthy people (Kuo et al., 2014; Davis and Smith, 2019). The specific functional brain region is stimulated by tDCS in the form of sustained low direct current (usually <2 mA) to influence the psychological capability of the human. It is worth noting that tDCS alters neuronal membrane potentials to affect spontaneous background cortical activity, and the enhancement of Hebbian plasticity caused by tDCS is proven to be relevant with the improvement on behavioral performance (Nitsche and Paulus, 2000; Kronberg et al., 2020). In general, anodal tDCS increases cortical excitability, whereas saturation under cathodal tDCS has an opposite effect (Nitsche and Paulus, 2000). Repeated stimulation by anodal tDCS induces stable changes in the neuroplasticity of the human motor cortex, which is considered the neurological basis of cognitive enhancement (Rioult-Pedotti et al., 2000; Monte-Silva et al., 2011). Previous studies involving the repeated application of tDCS focused on the clinical accumulative effects of tDCS in some specific diseases (Brunelin et al., 2012; Elsner et al., 2013; Ljubisavljevic et al., 2016; Thibaut et al., 2017). Present research suggests that performance on an attention test was promoted by anodal tDCS, indicating that single active tDCS can enhance an individual’s capability for attention (Coffman et al., 2012; Nelson et al., 2012; Roy et al., 2015). Additionally, repeated tDCS was considered to induce robuster effect than single tDCS according to clinical researches (Dumel et al., 2016; Yosephi et al., 2018). However, most studies have focused on the influence of single tDCS on the stimulation of attention among healthy people, and substantial evidence for the efficacy of long-term and regularly repeated stimulation by tDCS in improving attention is lacking.

Attention plays a significant role in mental function and has specific neurobiological bases (Posner, 2004). Posner and Petersen (1990) suggested that the concept of attention should be described as an attention network that contains three subnetworks (orienting, alerting, and executive control), which are generally considered independent of each other. The alerting network has been deemed to relate with the arousal systems of brain stem of the brain, which have the function of maintaining optimal vigilance over a period of time; the orienting network, with the capacity to select information from the sensory input, activates parietal cortex frontal eye fields; and the executive network means the effect of suppressing interference among responses and activates the anterior cingulate and medial prefrontal cortex (Fan et al., 2005; Min et al., 2016). The prefrontal cortex (PFC) was found closely connected with the attention function, and lesions of the PFC always produce a deficit in attention (Funahashi, 2001; Arnsten, 2006). More recently, evidence has emerged that the tDCS of the left dorsolateral prefrontal cortex (DLPFC) provides considerable benefits to the capability of attention. McIntire et al. (2014) found that 30 min of tDCS over the left DLPFC is more beneficial than caffeine for maintaining vigilance during sleep deprivation and that the cognitive benefit can last for a few hours. A total of 20 min of tDCS of the left DLPFC has also been found to produce greater attention function in healthy people (Miler et al., 2018). Therefore, it is appropriate to consider the left DLPFC as the targeted cortical area associated with attention in this study.

We aimed to explore the effect of repeated anodal HD-tDCS (12 tDCS sessions) on attention function during a long period of time (4 weeks) and to find the variation trend of attention function measured using an attention test. HD-tDCS can be used to apply current to the cerebral cortex with high density and precision and is more efficient than general tDCS (Kuo et al., 2013). The current intensity was set at 1.5 mA based on the maximum tolerance of the participants. It is worth mentioning that repeated application of HD-tDCS (1.5 mA, 20 min at a time) over a period of 4 weeks appears to be safe for participants based on what is currently known (Turski et al., 2017). Repeated non-invasive intervention on the cortex at suitable time intervals is thought to bring sustained benefit for participants (Monte-Silva et al., 2008). To maximize the benefit of the intervention, our experiment was designed so that each intervention day alternated with a rest day because sleep is considered necessary for enhancement of synaptic connections (Romcy-Pereira and Pavlides, 2005). An attention network test (ANT) was used as a measure of the efficiency of the attention network. ANT has been developed to measure the efficiency of the alerting, orienting, and executive networks according to reaction time during task performance (Fan et al., 2002). It is crucial for ANT to examine three attention subnetworks in a single task to not only obtain the efficacy of different attentions in the same task but also achieve an ideal result with the fewest relative trials (Fan et al., 2002; Fan et al., 2005). In addition, the color-word Stroop test (CW-Stroop) is a classical test that is often used to measure selective attention and inhibition (Treisman and Fearnley, 1969; Sugg and McDonald, 1994). Previous studies suggested that the same brain areas (anterior cingulate cortex and DLPFC) that underlie executive control of the attention network are activated during the Stroop task (Peterson et al., 1999; Vanderhasselt et al., 2009). We hypothesized that 4 weeks of anodal HD-tDCS stimulation might provide a successive benefit to the mental function of attention and that the pattern of improvement in the attention function in the experimental group would differ from that in the control group.

## Materials and Methods

### Participants and Design

#### Participants

A total of 39 right-handed healthy undergraduates (mean age = 21.15 years, SD = 1.78 years; 20 males and 19 females) completed this study. They were randomly assigned to an anodal group and a sham group at the beginning, and there were not significant differences in terms of age or educational background (Table 1). The results of 39 participants were analyzed at the end of the study, as 42 participants were initially enrolled in the study, but 3 volunteers dropped out before the baseline test due to conflicts between the experimental process and their course schedules. Vision and hearing were normal or corrected for all participants. This study was approved by the Ethics Committee of Tangdu Hospital (2014-03-03) and had been registered at ClinicalTrials.gov (NCT02420470, http://www.clinicaltrials.gov/). All procedures were conducted according to the principles set forth in the Declaration of Helsinki. All participants gave informed consent and were paid 4 weeks after the experiment.

**TABLE 1:** Demographic characteristics of participants.

	**Anodal group (*n* = 21)**	**Sham group (*n* = 18)**	***t* (or χ2)**	***P*-value**
Age (years)	21.57 ± 2.04	20.67 ± 1.28	1.63	0.11
Education (years)	15.57 ± 2.04	14.56 ± 1.25	1.84	0.07
Male/Female	11/10	9/9	0.23	0.63

*Values are expressed as mean ± SD. *P*-value and *t* (or χ^2^) were obtained by independent-samples *t*-test (or χ^2^ -test).*

#### Design

**FIGURE 1:**
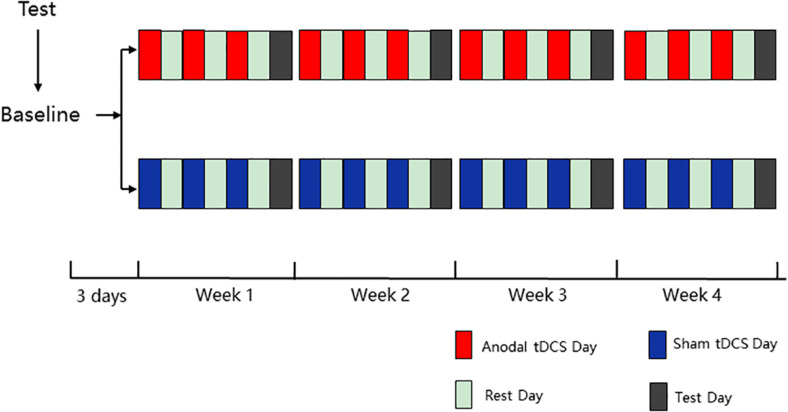
Overview of study design.

The experiment contained five phases (baseline, week 1, week 2, week 3, and week 4), and all 12 HD-tDCS sessions (3 sessions per week) were identical for each group in 4 weeks. In the baseline testing (3 day before the first intervention), all participants were asked to finish the ANT and the Stroop test, and there was a practice module lasting approximately 3 min before formal testing to ensure that the participants were familiar with the task. In the last four phases, during each week, three interventions were conducted every other day for the first 6 days, followed by ANT and the Stroop test on the last day (Figure 1). The intervention alternated with rest, which ensures adequate rest of the participants and the validity of the experiment. For the intervention, the anodal group was asked to accept anodal tDCS stimulation of the left DLPFC for 20 min, and the sham group was asked to undergo sham tDCS stimulation of the left DLPFC for 20 min.

### Cognitive Task

#### Attention Network Test

**FIGURE 2:**
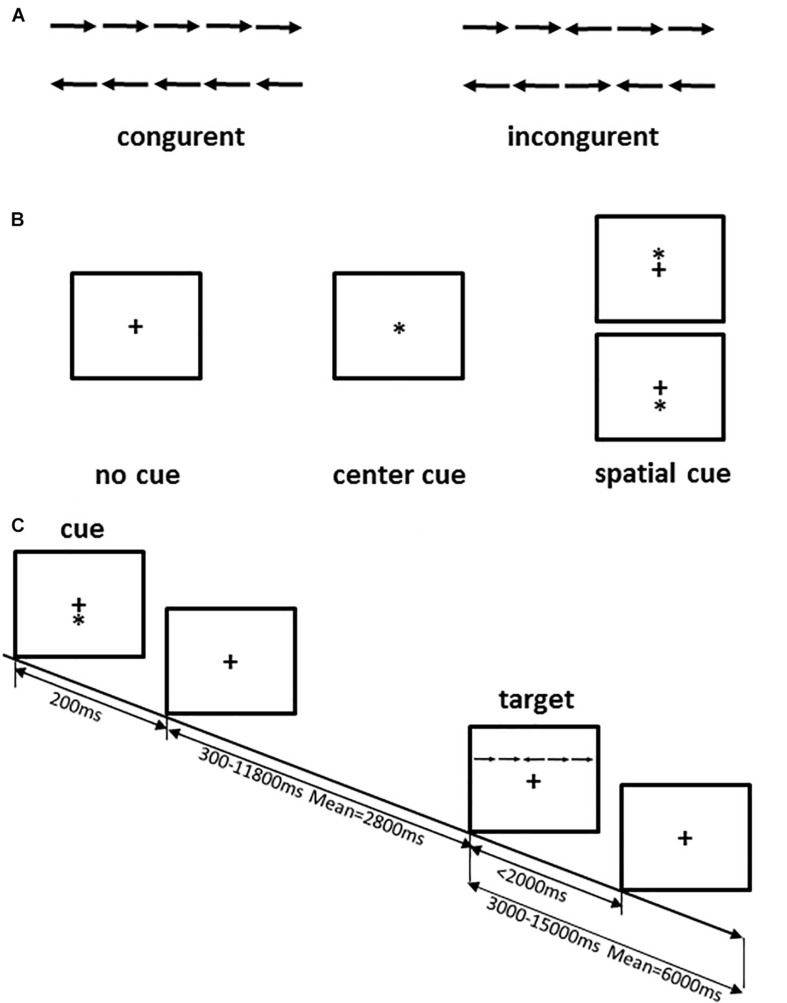
ANT procedure. **(A)** The four stimuli used in the present test; **(B)** the three cue conditions; **(C)** an example of the procedure. Adapted from Jin et al. (2005).

The ANT is widely used in research on attention because it provides an effective evaluation of attention networks based on objective behavioral indicators in the field of psychology. To improve the efficiency of the experiment and avoid the occurrence of mental fatigue in the participants, we selected a shorter version of the attention network activation used for the fMRI study (Jin et al., 2005). ANT is a cued-reaction time flanker task that was designed to measure the attention network (alerting, orienting, and executive functions) by detecting the reaction times of the participants. In this test, the stimuli are presented in the form of five horizontal black arrows that have left or right random directions. The arrows are cued by different cues, including a center cue, a spatial cue, and no cue. The stimuli were displayed simultaneously in the center of the screen, and participants were required to identify the direction of the central arrow flanked by four arrows and to provide a response as quickly as possible by pressing reaction buttons on the keyboard. A specific description of this version of the ANT is provided in Figure 2. In this version of ANT, there are 12 runs with 72 trials adding 4 buffers, and the sequence of 6 trials (3 cue conditions × 2 target conditions) is random. A cross is always shown in the center of the screen, and the cue condition (no cue, center cue, or spatial cue) is presented for 200 ms every time. In particular, to decrease the expectation effect, the interval time between two trials varies randomly from 3,000 to 15,000 ms.

#### Color-Word Stroop Test

**FIGURE 3:**
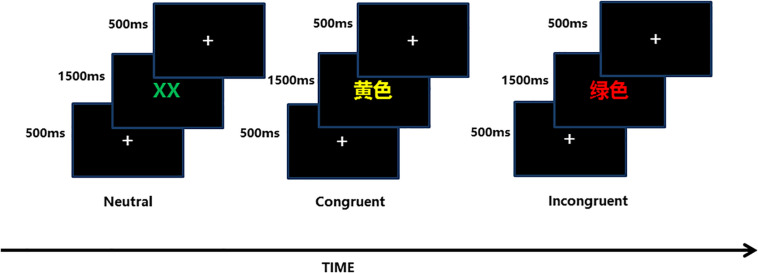
Each of the three conditions (neutral, congruent, incongruent) included 54 trials, and the sequence in which they were displayed was stochastic equilibrium.

The color-word Stroop test is a psychological test that is widely used to evaluate the Stroop effect, which is related to the interference of the dominant reaction with the non-dominant reaction. The task in our experiment was designed with three conditions: incongruent, congruent, and neutral, and the test stimuli included three Chinese words that represent different colors (“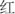,” “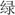,” “
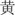
”) and a neutral stimulus (the English letter “X”) (Figure 3). The stimuli were displayed sequentially in random order, matching the three colors (red, green, yellow) randomly at equilibrium. The participants were required to identify the front color quickly and to verify the accuracy of each stimulus.

## Instrumentation

**FIGURE 4:**
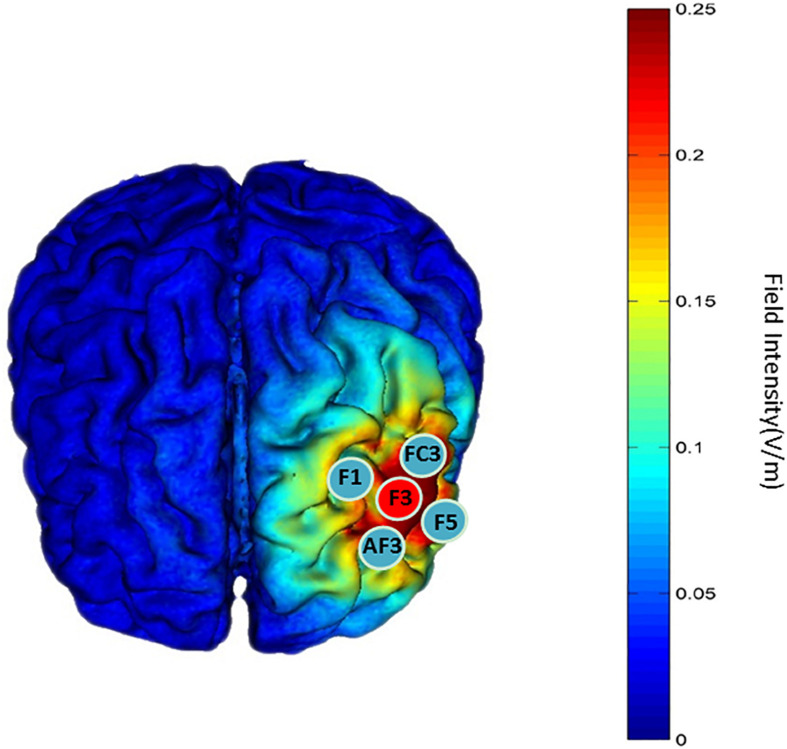
“Red patch” indicates the position of the anodal electrode. “Blue patch” indicates the position of the cathodal electrode. The theoretical current intensity at the cortex (left DLPFC) of the tDCS electrode array was calculated using Soterix HD-Explore.

A battery-powered constant-current DC stimulator (1300 A and 4 × 1-C3A, Soterix Medical, New York, NY, United States) was used to deliver 1.5-mA HD-tDCS stimulation in each intervention. The left DLPFC is the cortical area we targeted to enhance attention function by tDCS stimulation. The anodal electrode was placed on scalp location F3 according to the international 10-10 EEG System, and the four cathodal electrodes were placed over AF3, F1, F5, and FC3 (Figure 4); the theoretical current intensity to the cortex (left DLPFC) of our tDCS electrode array is shown in Figure 4. We placed conductive gel on the scalp under the hair to ensure connectivity before stimulating. The tDCS was applied at 1.5 mA for 20 min; this period included 30 s at the beginning of stimulation during which the current was ramped up to 1.5 mA and 30 s at the end for ramping down. Sham tDCS stimulation was applied at 1.5 mA for 1 min, including 30 s at the beginning for ramping up to 1.5 mA and 30 s at the end for ramping down. The participants were asked to disclose whether they had experienced a mood abnormality or fluctuation before or after stimulation, and their emotional stability was ensured. We always focused on the feelings of the participants during the intervention, and the stimuli were terminated if the participant was unable to tolerate the pain on their scalp. The participants reported no adverse effects except for slight tingling of the skin during current changes. In addition, we examined whether participants became aware of the sham condition after the experiment and found that almost all participants (89%) could not recognize the sham tDCS condition correctly.

### Analysis of the Effect on the Attention Network and CW-Stroop

In this work, the effect of three subnetworks was calculated according to the statistical analysis method (Jin et al., 2005). The effect of alerting was calculated by subtracting the mean RT observed under the center-cue condition from the mean RT observed under the no-cue condition. The effect of orienting was calculated by subtracting the mean RT observed under spatial cue conditions from the mean RT observed under center-cue conditions. The effect on executive function was calculated by subtracting the mean RT obtained under congruent conditions from the mean RT obtained under incongruent conditions. High alerting and orienting scores reflected improved capabilities of alerting and orienting. Conversely, low executive scores indicated enhancement of executive function. The Stroop effect was assessed by subtracting the mean RT observed under the neutral condition from the mean RT observed under the incongruent condition (Vitkovitch et al., 2002).

In our study, separate 2 (Group: active vs. sham) × 5 (Time: baseline, week 1, week 2, week 3, week 4) repeated measures ANOVAs tested for group differences in each task including multiple dimensions over time. For the ANOVAS, effect sizes were quantified by calculating the partial eta squared (pη^2^) (Cohen, 1973), and we followed the guideline proposed by Cohen to explain pη^2^ (0.01 (small effect), 0.09 (medium effect), 0.25 (large effect)). Additionally, we performed a Bonferroni-adjustment to limit the likelihood of Type 1 error, and used the corrected threshold of *P* = 0.05/3 for ANT and *P* = 0.05/4 for CW-Stroop. The data were presented as the mean ± SD. All analyses were performed using SPSS software v25.0.

## Results

### Baseline

The baseline differences between the anodal and sham groups in the three subnetworks of ANT under the three conditions and in the Stroop effect (RT_*Incongruent*_-RT_*Neutral*_) measured by the CW-Stroop were not statistically significant (Table 2).

**TABLE 2:** Mean reaction time of baseline on each condition of ANT and CW-Stroop, and Stroop effect in the anodal and sham groups.

	**Anodal group (*n* = 21)**	**Sham group (*n* = 18)**	***F***	***P*-value**
**ANT**				
Alerting	22.11 ± 44.00	33.60 ± 37.73	0.75	0.39
Orienting	7.81 ± 46.60	1.65 ± 27.14	0.24	0.63
Executive	90.96 ± 51.22	72.04 ± 32.07	1.83	0.18
**CW-Stroop**				
Neutral	536.86 ± 47.81	559.33 ± 55.23	1.86	0.18
Congruent	536.78 ± 57.69	548.55 ± 59.14	0.40	0.53
Incongruent	598.38 ± 60.24	633.81 ± 98.23	1.90	0.18
**Stroop effect** (Incongruent-Neutral)	61.52 ± 39.16	74.48 ± 59.82	0.66	0.42

*Values are expressed as mean RT ± SD.*

### Attention Network Test

**FIGURE 5:**
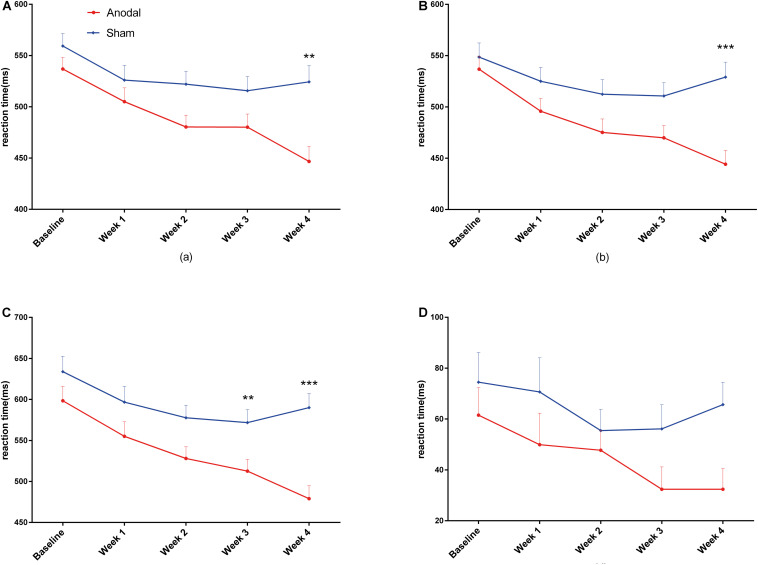
Variation in reaction time on executive under ANT from baseline to week 4 in the anodal group and the sham group. Error bars represent SEM for the change in reaction time. *P*-value is calculated by the comparison between two groups.

Repeated measures ANOVA on each dimension of the attention network was performed to analyze the effects of the three subnetworks; the descriptive statistics are shown in Table 3. There was no significant difference between anodal and sham groups in the alerting effect (main effect of time: *F*(4,148) = 0.26, *P* = 0.90, pη^2^ < 0.01; main effect of group: *F*(1,37) = 0.94, *P* = 0.34, pη^2^ = 0.03; interaction for time × group: *F*(4,148) = 1.33, *P* = 0.26, pη^2^ = 0.04). Similarly, there was no significant difference on the orienting effect (main effect of time: corrected by Greenhouse-Geisser, *F*(3.28, 121.20) = 2.31, *P* = 0.07, pη^2^ = 0.06; main effect of group: *F*(1,37) = 0.11, *P* = 0.74, pη^2^ < 0.01; interaction for time × group: corrected by Greenhouse-Geisser, *F*(3.28, 121.20) = 1.76, *P* = 0.15, pη^2^ = 0.05). As shown in Figure 5, the executive effect improved in both groups during the experiment at different levels (main effect of time: *F*(4,148) = 11.22, *P* < 0.01, pη^2^ = 0.23), and there was a significant effect of interaction regarding time and group (*F*(4,148) = 3.91, *P* < 0.01, pη^2^ = 0.10). The reaction time of the executive effect in the anodal group (mean change = 51.1 ms, SEM = 9.87) from baseline to the last phase decreased more than that in the sham group (mean change = 12.17 ms, SEM = 10.66). We conducted pairwise comparisons by different phases after Bonferroni adjustment in each group and found that the anodal group showed a significant benefit from week 1, earlier than in the sham group (Table 3). The improvements in executive at each of the 4 weeks compared to that at baseline were highly significant (*P* < 0.001) in the anodal group but not in the sham group (Table 3). There was a variation tendency from week 3 to week 4, after week 3, the mean reaction time on executive of the sham group began to increase, an opposite trend was observed in the anodal group, and the enhancement in the anodal group was marginally significantly higher than that in the sham group at week 5 (Figure 5). No consequential change in accuracy was observed in the data analysis, and the effect of the attention network was linked only to reaction time.

**TABLE 3:** Mean reaction time on three conditions of ANT from baseline to week 4 in the anodal and sham groups.

	**Baseline**	**Week 1**	**Week 2**	**Week 3**	**Week 4**
**Alerting**					
Anodal	22.11 ± 44.00	17.74 ± 37.88	25.68 ± 35.44	27.40 ± 45.35	24.13 ± 23.90
Sham	33.60 ± 37.73	45.23 ± 59.51	25.09 ± 34.56	20.37 ± 29.83	32.65 ± 50.79
**Orienting**					
Anodal	7.81 ± 46.60	18.15 ± 31.24	27.24 ± 33.73	18.66 ± 38.00	11.70 ± 20.02
Sham	1.65 ± 27.14	4.81 ± 31.28	12.05 ± 36.95	30.79 ± 36.78	24.33 ± 27.56
**Executive**					
Anodal	90.96 ± 51.22	44.86 ± 28.04***	46.16 ± 36.16***	48.11 ± 25.79***	39.86 ± 18.66***
Sham	72.04 ± 32.07	66.07 ± 38.21	50.91 ± 24.41	38.51 ± 37.74	59.88 ± 39.51

Values are expressed as mean RT ± SD. ***P < 0.001, compared to the baseline.

### Color-Word Stroop Test

**FIGURE 6:**
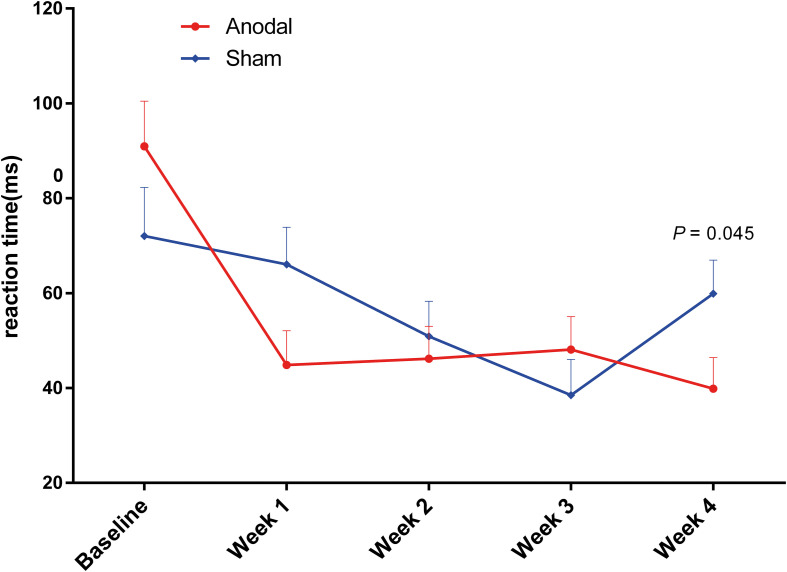
Variation in reaction time during CW-Stroop from baseline to week 4 in the anodal group and the sham group (**A** = neutral, **B** = congruent, **C** = incongruent, **D** = Stroop effect). Error bars indicate the SEM for the change in reaction time. **P < 0.01, ***P < 0.001, comparison between two groups.

For the CW-Stroop, repeated measures ANOVA was performed separately for reaction time under the three conditions (neutral, congruent, and incongruent) and for the Stroop effect. The descriptive statistics are shown in Table 4. For the neutral condition, the main effect of time (*F*(4,148) = 21.55, *P* < 0.01, pη^2^ = 0.37) and the interaction of group × time (*F*(4,148) = 4.94, *P* < 0.01, pη^2^ = 0.12) were significant, but the main effect of group (*F*(1,37) = 5.86, *P* = 0.021, pη^2^ = 0.14) was marginally significant. Similarly, the changes in mean reaction time were affected by time, group, and their interaction in the congruent condition (main effect of time: *F*(4,148) = 16.67, *P* < 0.01, pη^2^ = 0.31; main effect of group: *F*(1,37) = 6.64, *P* = 0.014, pη^2^ = 0.15; interaction of group × time: *F*(4,148) = 5.75, *P* < 0.01, pη^2^ = 0.13) and the incongruent condition (main effect of time: *F*(4,148) = 16.67, *P* < 0.01, pη^2^ = 0.35; main effect of group: *F*(1,37) = 6.64, *P* < 0.05, pη^2^ = 0.20; interaction of group × time: *F*(4,148) = 5.75, *P* < 0.01, pη^2^ = 0.10). For the Stroop effect, there were no statistically significant main effect of time (*F*(4,148) = 2.37, *P* = 0.06, pη^2^ = 0.06) and interaction of group × time (*F*(4,148) = 0.65, *P* = 0.63, pη^2^ = 0.02), and the main effect of group (*F*(1,37) = 2.37, *P* = 0.039, pη^2^ = 0.11) was marginally significant. The Bonferroni-adjusted comparison of the results obtained under the three conditions between baseline and weeks 1–4 is shown in Table 4; significant improvements under both congruent and incongruent conditions appeared earlier in the anodal group than in the sham group. Similarly, there was a variation tendency from week 3 to week 4 under the three conditions and the Stroop effect, after week 3, the mean reaction time of the sham group began to increase, and the improvements under three conditions observed in the anodal group were significantly higher than those observed in the sham group at week 5 (Figure 6). The accuracy rates were not further analyzed here because they were very high among all participants, and the interaction of group × time was not significant after repeated measures ANOVA.

**TABLE 4:** Mean reaction time on three conditions of CW-Stroop and Stroop effect from baseline to week 4 in the anodal and sham groups.

	**Baseline**	**Week 1**	**Week 2**	**Week 3**	**Week 4**
**Neutral**					
Anodal	536.86 ± 47.81	505.07 ± 64.89**	480.31 ± 54.43***	480.20 ± 66.56***	446.66 ± 48.94***
Sham	559.33 ± 55.23	526.05 ± 57.83**	522.14 ± 51.13***	515.67 ± 47.45**	524.36 ± 83.42
**Congruent**					
Anodal	536.78 ± 57.69	495.78 ± 55.90**	475.16 ± 67.91***	469.85 ± 60.41***	444.13 ± 51.37***
Sham	548.55 ± 59.14	525.07 ± 58.42	512.42 ± 49.54	510.73 ± 47.03	529.07 ± 72.68
**Incongruent**					
Anodal	598.38 ± 60.24	554.98 ± 84.88	528.05 ± 74.30***	512.58 ± 78.76***	479.05 ± 59.39***
Sham	633.81 ± 98.23	596.71 ± 77.19	577.59 ± 52.59**	571.82 ± 47.39*	590.05 ± 84.48
**Stroop effect**					
Anodal	61.52 ± 39.16	49.91 ± 58.58	47.74 ± 40.10	32.38 ± 43.28	32.39 ± 30.13
Sham	74.48 ± 59.82	70.66 ± 54.90	55.45 ± 29.44	56.14 ± 36.22	65.69 ± 44.02

Values are expressed as mean RT ± SD. Stroop effect was calculated by subtracting the mean RT of the neutral conditions from the mean RT of the incongruent conditions. *P < 0.013, **P < 0.01, ***P < 0.001, compared to the baseline.

## Discussion

This study examined the influence of application of repeated 1.5 mA HD-tDCS (20 min) to the left DLPFC (F3) on the attention function. Additionally, tDCS in our study is delivered “offline” and not “online” on account of the fact that the effect of “online” tDCS stimulation is influenced by the accumulative effect of repeated tDCS and immediate effect of current tDCS at the same time, so the behavioral outcomes become difficult to explain with clarity. The behavioral results, which were measured using the ANT and the color-word Stroop test, reflect the great benefits of HD-tDCS on the function of attention in the anodal group compared to the sham group, in agreement with previous studies (Gladwin et al., 2012; Andres et al., 2020). The ANT measures three discrepant and uncorrelated dimensions (orienting, alerting, and executive control); thus, the final result generated by the intervention is easily separable and analyzable (Fan et al., 2002). In the anodal group, we found that there were characteristic improvements in executive control but not in orienting or alerting; these results are similar to those reported in a recent study (Miler et al., 2018). The multinode framework was developed recently; it observes the effect of the targeted brain network and task load and is different from the traditional dual-polarity framework, which is less suitable for predicting the effect of tDCS (Rodrigues De Almeida et al., 2019). According to the multinode framework, the different changes observed in the three subnetworks should reflect two aspects: (A) the left DLPFC, the cortical area targeted in our experiment, is considered an essential part of the executive control network and differs from the orienting and alerting networks based on imaging evidence (Carter et al., 2001; Fan et al., 2005; Petersen and Posner, 2012); additionally, studies have shown that stimulation of the right parietal cortex or F10 positively modulates orienting and alerting (Coffman et al., 2012; Roy et al., 2015); (B) the fMRI version of the ANT is a highly efficient simplification, but may be less relevant to the task of functional targeting; therefore, based on previous research on the modulation of executive control, we added a supplementary task, the CW-Stroop test, to ensure sufficient task load for measuring executive control for attention and selective attention. It is worth pointing out that positive behavioral outcomes were observed in all three conditions, a result similar to the results of a previous study (Loftus et al., 2015); however, the improvement on Stroop effect was not significantly different between two groups, so it is robust to consider that the current results just indicate enhancement on psychomotor efficiency.

The improvements in executive control in the anodal group as measured by ANT were significantly different compared to baseline in each of the 4 weeks of the experiment, and these changes occurred from week 1, in contrast to the results observed in the sham group. The same variation tendency was found for congruent and incongruent conditions in the CW-Stroop. In addition, there was an interesting finding, as shown in Figures 5, 6, in that the performances of the sham group on ANT (only executive) and CW-Stroop began to return to the baseline level at week 3, but the benefits of anodal tDCS were maintained until the last week (week 4); the improvements on executive and three conditions of CW-Stroop in the anodal group were significantly higher than those in the sham group at week 4. At least, according to the evidence from our experiments, repeated anodal tDCS has the potential to eliminate the variation that attention function restores to its initial level. A recent study suggested that repeated anodal tDCS (10 sessions) induces cumulative effects on the cognitive performance of patients with brain injury (Ulam et al., 2015), and significant superiority in the anodal group appeared only in the later period in the experiment; therefore, we speculate that a cumulative effect of anodal tDCS is a possible reason for the specific variation in attention function in our study. Furthermore, late LTP-like (late long-term potentiation-like) plasticity is pivotal in perceiving and learning, and it is considered a biological explanation for the permanent and steady influence of repeated anodal tDCS (Feldman, 2009; Monte-Silva et al., 2012). We hypothesized that the change in late LTP-like plasticity is a potential and probable basis of the neurological changes observed in our study; however, this point should be addressed in further experiments.

To measure the attention function at each phase of experiment, it is necessary to use the ANT and CW-Stroop test repeatedly, and the changes in the sham group were significant compared to baseline during the later experimental period, possibly due to the learning effect caused by repetition of the cognitive task. Of course, the possibility that a placebo effect occurred in the sham group should be considered. The study results are persuasive since it has a similar sample size to other studies, but it would be more precise to increase the sample size in a future study. In particular, the proportions of males and females in each group were as balanced as possible to avoid gender difference. The rate of improvement in attention is gradual and slow; for this reason, we think the ceiling effect was reached in the two tasks for participants with high cognitive function. The outcome of our study provides experimental evidence for changes in attention function caused by repeated tDCS and provides a feasible explanation for the interaction between the cortical network and current stimulation at the cellular level. However, we did not conduct tDCS of other targeted cortical areas to measure a possible modulatory effect on the orienting and alerting networks. The detection of mechanisms related to the long-term effect of anodal tDCS on attention through imaging and physiological techniques clearly makes sense, and further studies should focus on seeking evidence through functional magnetic resonance imaging (fMRI), functional near-infrared spectroscopy (fNIRS), or electroencephalography (EEG).

## Data Availability Statement

All datasets presented in this study are included in the article/supplementary material.

## Ethics Statement

The studies involving human participants were reviewed and approved by the Ethics Committee of Tangdu Hospital. The patients/participants provided their written informed consent to participate in this study.

## Author Contributions

HL, QL, ZG, GZ, YZ, and SW designed and conducted the study, including recruitment of participants, data collection, and data analysis. SW provided experimental equipments. HL and QL prepared the manuscript draft with important intellectual input from ZG, GZ, and YZ. XZ provided editorial support during preparation of the manuscript. All authors approved the final manuscript. All authors contributed to the article and approved the submitted version.

## Conflict of Interest

The authors declare that the research was conducted in the absence of any commercial or financial relationships that could be construed as a potential conflict of interest.
